# Resonating moments: Exploring socio-material connectivity through artistic encounters with people living with dementia

**DOI:** 10.1177/14713012211039816

**Published:** 2021-10-24

**Authors:** Lilli Mittner

**Affiliations:** Artful Dementia Research Lab, Centre for Women's and Gender Research, 100708 UiT The Arctic University of Norway, Tromsø, Norway

**Keywords:** situated art intervention, socio-material connectivity, sound and music, aesthetic analysis, arts-based research, new material feminist theories

## Abstract

In this article, I introduce insights from new material feminist theories into the understanding of connectivity on the basis of an aesthetic analysis of artistic encounters with people living with dementia. I draw on data from a situated art intervention conducted within the Resonance Project at a residential care home in Northern Norway where researchers, artists, health-care professionals, people living with dementia and family members came together in co-creative music sessions. I analyse two resonating moments from the sessions by way of an abductive process, oscillating between theory, written notes, video recordings and my own embodied experiences in the field. I discuss the ways in which materiality, listening and the group matter when it comes to our ability to connect during the sessions. Based on these findings, I conclude that the notion of socio-material connectivity provides an entrance point for studying different ways of relating to people living with dementia and enquiring into relations that matter.

## Introduction

The arts are increasingly receiving attention from researchers, artists and policy-makers alike as regards improving health and well-being (Fancourt & Finn, 2019). A growing body of arts-based research in dementia studies has elaborated on how to meaningfully connect through creative expressions such as music, theatre or fine arts (e.g. Boydell et al. (2016); Kontos et al. (2017); Zeilig et al. (2019)). Art interventions are acknowledged as facilitating social connectivity (de Medeiros & Swinnen, 2018, p. 68) and providing a unique potential for exploring and developing new understandings of human relations. However, most of the research ‘fails to capture the quality of engagement; it provides the “who” and “what” but not the “what does it mean?”’ (de Medeiros & Swinnen, 2018, p. 68).

Health-care staff, people living with dementia and their next of kin all call for recognition of the need to connect. Health-care professionals report on their need to connect in order to provide care in respect of ‘the most basic physical tasks such as eating, toileting, bathing, and walking’ (Kontos & Martin, 2013; see also Armstrong and Armstrong (2003) and Adams and Gardiner (2005). People living with dementia have shown a desire to connect with themselves, peers and the environment (Keyes et al., 2016; Zeilig et al., 2018). Next of kin have reported on their needs in various qualitative studies (Hazzan et al., 2016; Johnston et al., 2017; Larsen et al., 2019). The human ‘quest for resonant relationships’ (Rosa, 2019) finds broad agreement across academic disciplines (Baumeister & Leary, 1995; Bresler, 2006; Cornwell & Waite, 2009), although Joanna Latimer has signalled caution in respect of the discourse according to which everyone ‘needs and wants connection’ (Latimer, 2018, p. 839).

Attempts to connect rely often on a search for the ‘self behind the disease’ (Kitwood, 1997) in order ‘to value who the person is’ (McDermott et al., 2014, p. 706). A central assumption is that we are better able to connect if we know who we are. However, attempting to build strategies to connect with people living with dementia on memory, rationality and cognition often results in frustration, sorrow and a feeling of loss. Many attempts to connect assume a coherent mind and aim at retaining ‘normal behaviour’ in the sense of the ‘autonomy and individuality that underpin person-centeredness’ (Kontos et al., 2017, p. 183; see also Dupuis et al. (2012)).

Connectivity is mainly conceptualised as a ‘dynamic interplay between individuals’ (Campo & Chaudhury, 2011). Non-verbal communication and environmental and physical factors such as place (Moore, 1999) have received occasional but not systematic attention within the analysis of social interaction. However, what matters is the connection with both ‘social and material worlds’ (Hillman & Latimer, 2017, p. 2). Broadening the focus from social connectivity (Cornwell & Waite, 2009; de Medeiros & Swinnen, 2018) to socio-material connectivity allows the consideration of sound, space and other more-than-human agents of ‘the larger world’ (Basting, 2020, p. 101). The materiality of everyday artefacts plays an important role in the practice of care and is important to our relations, our ability to connect and the ways we experience the world around us (Buse & Twigg, 2018). In a music session, this means that not only people but also musical instruments and sound become a part of entangled relationships.

The purpose of this article is to explore connectivity more broadly by asking who and what are connecting and how can these connections be understood? I reflect upon these questions on the basis of mutual aesthetic encounters with people living with dementia. My findings are based on a situated art intervention conducted within a residential care home in Northern Norway. After a short presentation of the Resonance Project, I will introduce my theoretical framework, which is based on new material feminist theories and the concept of intra-action that is central to my work. I then present two resonating moments that occurred in the music sessions and discuss fundamental shifts in my thinking practices based on those moments. I conclude that the notion of socio-material connectivity provides an entrance point for studying different ways of relating to each other and enquiring into the relations that matter.

### The Resonance project

In 2019, I developed a situated art intervention at a residential care home in Northern Norway together with artists, health-care professionals, people living with dementia, and family members. In my role as a research artist, I intervened in everyday practices at the residential care home and became part of the emerging relations and aesthetic experiences (Dewey, 1980) that I was studying. The research and intervention project comprised 14 visits to the residential care home: five fieldwork days (from 11 a.m. to 3 p.m.) and four co-creative music sessions in the autumn of 2018 and five co-creative music sessions in the spring of 2019. The music sessions were arranged weekly to begin at 11:30 a.m. and lasted about 60 min, depending on the needs of the group. There were between 7 and 10 of us, and we would gather in a small activity room, sing, talk about music-related and other topics, and experiment with instruments. All these actions can be seen as part of the larger concept of musicking, in which a variety of music-related actions are involved in what is perceived as ‘music’ (Small, 1998).

Sound and music triggered some surprising and unexpected moments. For example, people who were sitting quietly next to each other in the dining room or doing structured activities would start to talk to each other and interact, both verbally and non-verbally, during the sessions. An effect of this is that relations, people and meaning transformed.

The activities were in the format of leisure activities (Genoe & Dupuis, 2014) and built on play (Swinnen & de Medeiros, 2018) and imagination (Basting, 2020). Rather than seeking normalisation, the aim was to find innovative ways to connect by engaging with sound and music and requiring abilities that were not necessarily rational. Hence, all of the activities that unfolded were highly improvised and only to a very limited extent pre-structured. Openness to the unexpected permitted the humbleness that precedes the unknown. Only time and place were fixed. Even the room in which we gathered was negotiable in the beginning. Routines and trust developed as the members of the group got to know each other. This open-ended approach was important for making it possible to remain responsive to the unexpected. This situated art intervention enhanced the likelihood of relying intuitively on mutual understanding and collectively building a way of being together.

### Ethical considerations

Those with dementia and those without dementia were treated equally and everyone was seen as an active citizen who could make an equal contribution to the creative activities of the sessions within relational aesthetics (Bourriaud, 2002). I gave the participants a broad outline of the project by phone or face to face during the 2-month fieldwork period that took place in advance of the music sessions. Legally authorised representatives (next of kin) gave consent by proxy for the residents who could not give consent themselves. Consent was monitored and updated throughout the research process, and there was an option to withdraw from the sessions at any time. This included an ongoing option to take part or not take part on a day-to-day basis. Before each session, the care home’s activity leader and I told the residents that participation was voluntary and that one could leave, or ask for assistance to leave, at any time. Beyond legal consent, the project’s activities were conducted in keeping with relational ethics that ‘take into account the immediacy and complexity of the particular situation and our moral responsibility within it’ (Austin, 2008). Decisions regarding when to change action, when to interrupt, when to stop and when to proceed were always made collectively by people while taking into account time, spatiality and materiality. On occasion, we had to interrupt a session because someone left, sometimes because the songbook was turned upside down, sometimes because it was time for lunch. For people who need structure and predictability, there was a potential risk of psychological distress from the high level of uncertainty that was crucial to the project design. However, due to the self-selection recruitment process, the people who stayed and re-joined the group on a weekly basis were open to the high degree of openness connected with what the group might create together. The overall benefit of the situated art intervention was considered by the group to be greater than its risks.

### Theoretical framework

The subject of this article is situated within a larger inter- and transdisciplinary project for rethinking dementia beyond human individual loss (Artful Dementia Research Lab, 2021). The prevailing discourse on dementia is dominated by a ‘deficit model’ (Latimer, 2011) that affects care practices and the way that people living with dementia are perceived and perceive themselves. Feminist scholarship enables us to view what makes life worth living in different ways and offers promising approaches to encountering various dilemmas in respect of what it means to be human. Morten Hillhaard Bülow’s and Marie-Louise Holm’s work on queering ‘successful ageing’ (Bülow & Holm, 2016), Joanne Latimer’s reading of Gladys Wilson (Latimer, 2018) and Dragana Lukić analysis of multiple ontologies of Alzheimer’s disease (Lukić, 2019) are a few examples how feminist scholarship can help us to rethink what a life with dementia can become.

New material feminist theories allow us to think ‘agency increasingly as not merely a human affair’ (Tiainen, 2018, p. 108). As is everything else, relations are in a constant state of becoming (Nussbaum, 2013). If neither subject nor object positions are fixed but enacted (Haraway, 2016; Lukic, 2019; Mol, 2008), then meaning is not something that exists a priori but rather something that is constituted in relations (Bennett, 2010; Grosz, 2012; Alaimo & Hekman, 2008). As Ann Therese Lotherington has pointed out, rethinking dementia through feminist material perspectives creates situations ‘in which the distinction between those with and those without a dementia become irrelevant, and where a connectivity can emerge through common aesthetic experiences’ (Lotherington et al., 2018). Hence, understanding dementia as a dynamic, collective process instead of as a static, individual affair suggests major conceptual changes in future care and society (Hillman & Latimer, 2017; Latimer, 2018; Puig de la Bellacasa, 2017; Shakespeare et al., 2017).

The concept of intra-action is promising as regards rethinking what happens when we connect. Physicist and feminist scholar Karen Barad suggests that relations are transformed and reconfigured, based on what happens not only between, but also within, agents of different kinds. Transferring this to social relationships with people living with dementia, it suggests that individuals do not pre-exist their interactions; rather, ‘individuals emerge through and as part of their entangled intra-relating’ (Barad, 2007, p. IX). The reader may think at this point of two children swinging a skipping rope between them. Only if they find a rhythm and go on with the flow will the rope move equally between them and their action become meaningful. The concept of intra-action, as proposed by Karen Barad, can serve as one entrance point for understanding mutual relations beyond a linear connection, as an ongoing process that takes into account that we are transforming all the time.

The notion of resonance safeguards further against the danger of thinking of connectedness as similitude and furthers ‘help[s] us to get away from an idea of mutuality that requires that humans and non-humans have to completely attend one to the other or share the same purpose’ (Latimer, 2013, p. 93). Experiencing resonance in music has been described as attunement: as two or more entities that are tuning in to each other in the sense of becoming with each other (Coomans, 2016; DeNora, 2016; Gerber et al., 2018). Some researchers suggested the possibility of ‘resonating in dissonance’ (Lindvang et al., 2018, p. 14). Resonance further relates to the notion of flourishing (McCormack & Titchen, 2014; Ryff & Singer, 2003), which means ‘to participate fully and voluntarily in social connectedness with another in a way that gives and provides personal meaning’ (de Medeiros & Swinnen, 2018, p. 70). The notion of resonance provides meaning beyond the human individual and shifts the focus to something that changes between us. Resonance connects differences; it results in something greater than the sum of its parts and relates to growth (Patterson & Wolverson, 2016, p. 152). Hence, resonating moments are beautiful moments that can serve as a unit for analysing socio-material connectivity.

## Methods

Situated art intervention is an ‘open ended practice’ (Barad, 2007, p. 146) that aims to intervene in specific practices and simultaneously analyse them (Zuiderent-Jerak, 2015). This affects the role of the artist, who becomes decentred beyond an individual master narrative and within the broader context of everyday creativity and more mundane forms of arts-based practice in the community (Bellass et al., 2018). Furthermore, engaging residents as audience, participants or co-creators can have fundamentally different effects in terms of both aesthetics and ethics (Kontos et al., 2017; Artful Dementia Research Lab, 2021b). For example, interpreting even the slightest bodily expression as an aesthetic expression may lead ultimately into a ‘reciprocal creative game’ (Kontos et al., 2017, p. 14) in which ‘creative action is not an intellectual operation but rather […] arises from practical involvement’ (ibid.).

The two resonating moments presented in the following sections were extracted through an abductive analytical process that oscillated between theoretical framework, embodied practice and closeness to textual material such as field notes, logbook writings and video recordings. The aesthetic analysis as ‘embodied creative methods of inquiry’ (McComback & Titcher, 2014) was discussed by a team of three researchers, of whom two had an artistic background and one had a social science background. Their involvement in the sessions as research artists as well as by taking field notes was also part of the analytical process (Pink, 2009). Hence, the aesthetic experiences of connectivity described in the following section belong neither to the research artist nor to the residents exclusively, but rather to all of those who became entangled in the process of meaning-making through listening and reading (Bresler, 2006).

### Rrrrr and the guiro

The group sits together, chatting and listening to a resident playing Western classical piano music in the background while Susan (all names are fictive) holds a guiro and strikes it occasionally. We are in a listening-and-waiting situation. All of a sudden, Marleen makes a sound with her tongue, a low and deep rolling ‘rrrrr’. Susan, who is Marleen’s daughter, suggests this as a sign that her mother is freezing and arranges a blanket for her. I am listening, too, and after a while, I gest hands ask for the guiro that Susan was holding. ‘Listen, the guiro makes a similar sound,’ I say. Marleen listens, watching the guiro. The sound goes along with the tremolo of her hands. Holding her trembling hands is like holding the sound. I want to touch her hands – not to keep her calm as an act of normalising but as a way to connect with her expression through touch. The guiro makes a sound that resembles what Marleen does with her tongue.

Later, Susan expresses embarrassment through her body language when Marleen performs the ‘rrrrr’ in front of the group. She even puts words to what her body mimicked: ‘Now you have to stop Mum!’ The video recording reveals that Susan is slightly uncomfortable. Her discomfort may originate in her mother’s acoustic contribution, which is neither language nor any other expression found in Western music’s vocal aesthetics. I am also affected by Marleen’s ‘rrrrr’, but in a different way. Without any words, but only through the sound of the guiro, I ask Marleen what Basting (2020) conceptualises as a ‘beautiful question’ in the sense of ‘What does joy mean to you?’ Marleen looks at me, right into my eyes, and I feel a sensation of resonance. Suddenly we are connected.

In that moment, something changed in the room. Marleen answered my sounding question in her way: ‘Rrrrr.’ It was as if she were saying: listen to me, this is what joy means to me. And then we were making the sound together. Susan looked at us and smiled. I went into a short conversation with Marleen and her way of communicating through sound-making. She responded and we were musicking for a moment. The other group members encouraged us by following our actions (Fieldnotes 6.3.2019).

The materiality of the guiro, its sound, the group and our way of listening give rise to this moment. It is a moment of re-interpretation that allows Susan to see her mother in a different light. She reports after the session that it has been a long time since she has seen her mother in this state, and that it is good to see her enjoying the sessions.

Marleen transformed from a resident who appeared to me as fragile, poor, and weak into a strong, dedicated woman who presented her unheard sound to us. Everyone watched and listened to Marleen and the guiro. She seemed to appreciate the attention and the feeling of contributing something valuable to the group. The group’s reactions seemed to give her confidence. This shared moment established an expression within the group that made it possible for Marleen to contribute on her terms and feel that she was respected in her way of being together with us (Fieldnotes 6.3.2019).

It is not only Marleen who transformed in that moment but also Susan, whose perception of her mother shifts. The whole transformation occurs through sound, touch, smile, sight and laughter. Later, Marleen uses the sound to get the attention of the group when we meet outside the activity room. She uses the expression when I turn to her or when I pass by. As the group sessions proceed, other residents mention Marleen and ‘her sound’, even if she cannot stay with us. This shared moment of meaning-making, which involved the whole group, has had some lasting effects.

### The triangle moment

I am holding the white string of the triangle and Anne takes the role of player of the shining and dangling metal. We are all part of that shared moment. By holding the triangle up in the air towards Anne, I invite her into creative expression. Anne uses the baton, which becomes a wand to express her imagination. It is in the sense of a time-matter cut that we can hear the sound even before it is produced. The 440 HZ frequency of the shining metal spreads in the room and transformed into social resonance.

The triangle we used had a smooth and high vibrating sound that made people listen attentively, or which at least made them take notice. Before, during, and after the room being immersed in the triangle sound, everyone was listening and waiting with curiosity for what might happen next. The sudden sound became an agent itself, giving us the feeling of being here and now. Right after the sound disappeared, a short moment of contemplation followed. Anne and I smiled at each other without saying anything (Fieldnotes 27.3.2019).

It is a moment of becoming-with before clapping, laughing and amazement fill the soundscape. The prominent sound, together with the shape of the polished, sparkling, shining, delta-shaped silver metal are of crucial importance to this moment happening.

A similar moment occurs during another session when Marleen accompanies her daughter Susan and granddaughter Vivian. It appears to be of great importance to Susan that her mother has the privilege of closing the session. She urges Marleen to play the triangle properly by correcting her, for instance, by saying: ‘You have to do it this way!’ Using the triangle differently is not accepted. Marleen waits. The group waits. On the video recording, it seems as if Marleen was conducting our waiting. It is her turn to decide when to proceed. Nobody knows what will happen next. Will Marleen reject the baton and put it aside? Will the triangle stay silent or will there be a high sound – once, twice or many times, like a bell ringing? The whole group is connected in expectation, and it seems to me that Marleen is aware of the power she is holding in her hands. Suddenly, Marleen uses the baton with such confidence that it releases all of our expectations. Her family is proud that she has accomplished a task. The group applauds. Marleen smiles brightly with an expression of agency.

As the series of sessions proceeds and confidence and mutual trust develop, playing the triangle becomes a sort of initiation rite for our group. The triangle is explicitly not a pre-structured element brought by the research artist to the session but a structural element created by this specific group and in that specific place. It marks the transition from a mode of individual (dis)abilities to a mode of becoming with each other and co-creating our interpretation of Norwegian songs together.

## Discussion

The music sessions can be seen as an ongoing attempt to move skipping ropes between us. In order to find a mutual, collective, concerted, conjoined and shared rhythm, we were all dependent on instruments, bodies, sound and space. As music sociologist Tiia DeNora frames it, a variety of items were ‘involved in the production of [the] social “experience” and in the mobilization of cultural and aesthetic materials for being, doing and feeling’ (DeNora, 2001, p. 176). In the triangle moment, both the haptic material itself and the sound that spread in the room and in our bodies made us relate differently to each other. Shape and size made the triangle attractive for the group members to use. Any of us might have touched a triangle before, and there was a shared expectation about what was going to happen. When the triangle sound reached our ears, the room was filled with feelings of satisfaction, confidence and release after a moment of uncertainty. Even though the sound of the triangle may have been meaningful to each member of the group in different ways, it created a socio-material connection between us. A similar mechanism was found in the situation with the guiro and exemplified the ‘embodied nature of engagement […] involving the imagination, art and music’ (Kontos et al., 2017, p. 12). Since the aim was not ‘to bring someone with dementia back to the “normal” reality’ (ibid.), but rather ‘co-constructing an imaginative scenario’ (ibid.), the guiro was not just a tool for communication but also became an integral part of what was resonating.

Not only the instruments but also the sound transformed in meaning. Suddenly, the ugly ‘rrrrr’ became a beautiful sound that made sense in relation to the guiro. The ‘rrrrr’ was no longer perceived as the signature of a disease but as a creative expression that triggered an artistic response and played a central role within the ‘reciprocal creative game’ (Kontos et al., 2017, p. 14). In my role as the research artist, I tried to remain with the resonating moments as they occurred. In a different situation, as a result of her discomfort, Susan might have stopped the ‘rrrrr’ made by Marleen. Likewise, the activity leader might have stopped it due to time pressures or the perceived meaninglessness of the sound. Within the setting of the co-creative music session, however, we all gained the competence and privilege of waiting, listening and collectively making sense of the sound. The act of listening pushed the boundaries of what might be possible (Basting, 2020). By framing the sound with the instrument, demonstrating the material, and not just saying but replaying, the material created a meaningful moment of re-interpretation.

Making sense out of chaos is an audacious process. Residents, family members, researchers, artists, health-care professionals: we all had to follow carefully in order to make sense of what was going on. And we all played a crucial role in shaping the sessions. None of us could know in advance when a resonating moment of shared experience, shared ownership and shared attention might occur. As a result, something changed not just between us but also within us and had a lasting effect. Weeks after those moments occurred, the group was referring to them, and it felt like we could all invite each other ‘back to that moment again and again’ (Basting, 2020, p. 119). The relations that unfold in sound and music transformed dementia into a dynamic process. It was no longer important to be able to keep a conversation going, laugh at the ‘right’ moment, or remember correctly. The new ‘normal’ was re-defined by the group beyond person-centeredness, autonomy and rationality.

Within the relational model of dementia, the community is important to how we experience dementia (Ryan & Nolan, 2016, p. 221). The group affects how we relate and who we become, through and as part of our ‘entangled intra-relating’ (Barad, 2007, p. IX). Had it not been for the embarrassed daughter, Susan, I would not have tried to transform the sound. Or if there had not been an appraisal by the group of the triangle sound, it would probably just have been a triangle sound without any further meaning. The mutual aesthetic experiences during these sessions stand in contrast to what I’ve learnt from my beloved grandfather, who always feared dementia and communicated his fear long before he received the diagnosis. The way the group intra-acted during the music sessions made me understand that a life with dementia does not have to mean individual human loss; such a life can also be enacted in joy, creativity, spontaneity and open-endedness (Mittner et al., in print).

## Conclusion

On the basis of the aesthetic analysis of the two resonating moments, I have discussed who and what is connecting and how to understand those connections. I explored how materiality, listening and the group matter to the connections described above and how the findings affect my thinking practices as a researcher, artist and granddaughter of a man living with Alzheimer’s disease. Going beyond the human and conceptualising socio-material connectivity as an ongoing process between and within enabled me to better understand that the ability to connect resides within an ongoing intra-action. I understand how connections are based on changes within and in between. In the moment we connect, we are already transformed. Instruments, bodies, sound and space are enabling and are simultaneously part of the connections that matter. My findings further highlight that the situatedness of a moment in which we connect needs special attention. Bringing feminist and aesthetic perspectives into dementia research and the enactment of dementia may fundamentally affect our understanding of what it may mean to live with dementia.

### Limitations

Situated art intervention is a complex apparatus that allows one to produce and simultaneously enquire into moments that matter. Building a creative space that allows for collective enquiry into mutual relations is time-consuming and requires a high degree of trust. Sitting back and waiting was an often limited yet practical constraint and everyday practice at the site.

Resonating moments can be reported to a limited extent by means of academic vocabulary and within a linear written research paper. The trustworthiness of the science reported here depends equally on the reader immersing themselves in the embodied experience of resonance by reading (Crowhurst & Emslie, 2020). In addition, people living with dementia were part of the data creation and the initial analytical phase at the site; however, the article was not co-written with any of those who became entangled in the resonating moments. The visual material from the video recording of the moments reported, analysed and discussed could not be included in the article due to ethical and aesthetic constraints. Hence, the possibilities and challenges of situated art intervention research as an arts-based research method need to be further investigated.
